# A Complex Network Theory Approach for the Spatial Distribution of Fire Breaks in Heterogeneous Forest Landscapes for the Control of Wildland Fires

**DOI:** 10.1371/journal.pone.0163226

**Published:** 2016-10-25

**Authors:** Lucia Russo, Paola Russo, Constantinos I. Siettos

**Affiliations:** 1 Combustion Research Institute, Consiglio Nazionale delle Ricerche, Naples, Italy; 2 Department of Chemical Engineering, Materials and Environment, Sapienza University of Rome, Rome, Italy; 3 School of Applied Mathematics and Physical Sciences, National Technical University of Athens, Athens, Greece; Albert-Ludwigs-Universitat Freiburg, GERMANY

## Abstract

Based on complex network theory, we propose a computational methodology which addresses the spatial distribution of fuel breaks for the inhibition of the spread of wildland fires on heterogeneous landscapes. This is a two-level approach where the dynamics of fire spread are modeled as a random Markov field process on a directed network whose edge weights are determined by a Cellular Automata model that integrates detailed GIS, landscape and meteorological data. Within this framework, the spatial distribution of fuel breaks is reduced to the problem of finding network nodes (small land patches) which favour fire propagation. Here, this is accomplished by exploiting network centrality statistics. We illustrate the proposed approach through (a) an artificial forest of randomly distributed density of vegetation, and (b) a real-world case concerning the island of Rhodes in Greece whose major part of its forest was burned in 2008. Simulation results show that the proposed methodology outperforms the benchmark/conventional policy of fuel reduction as this can be realized by selective harvesting and/or prescribed burning based on the density and flammability of vegetation. Interestingly, our approach reveals that patches with sparse density of vegetation may act as hubs for the spread of the fire.

## Introduction

Efficient wildland fire control is one of the most challenging and important problems in ecology [1]. On the one hand, fire is an essential renewing contributor of the ecological cycle. On the other hand, wildfires have been the cause of irreversible environmental and socio-economic damages. Fuel management treatments have been extensively applied at the local scale, but they have had a limited influence on the evolution of wildfires at the landscape scale [2, 3]. At this scale level, experimental work is prohibitory, and the majority of previous studies on the spatial distribution of fuel management activities have been theoretical [2]. Observations on real wildland fire cases have evidenced that fire size and severity can be mitigated by proper design of treatments such as fuel segmentation and prescribed burning [3–6]. Hence, organizations like the USDA Forest Service, in their fuel managements plans, consider spatial fragmentation of the fuel crucial [7]. The problem is how to spatially distribute the fuel management activities across the landscape. Factors such as weather/climate conditions (wind field, air humidity and temperature), characteristics of the distributed local fuel (type and structure of the vegetation, moisture and density), landscape/earth characteristics (slope, fragmentation and natural barriers) as well as fire-suppression tactics are considered to be key elements [3, 8–14]. The conventional/empirical approach is that of selective fuel reduction, i.e. the selective cutting/harvesting and/or prescribed burning to reduce ground fuel at specific locations, based primarily on the density and flammability of the vegetation [15–17]. Recently, the US Forest Service started a massive forest thinning project for preventing devastating forest fires, especially in the Southwest [18]. However, special care has to be taken not to disturb the landscape in order to maintain the structural and spatial heterogeneity of the forest including the preservation of wildlife habitat [19, 20].

There is a growing interest of developing and implementing rigorous mathematical methods to help designing better fuel treatments. The base of these approaches is the adoption of accurate and efficient simulation models. At the small scale, theoretical studies on the spatial distribution of fuel managements activities have shown that a random or compartmented distribution of fuel management activities reduce the spread rate of the fire when dealing with large portions of the landscape [21, 22]. It has been also shown that regular patterns such as parallel stripes work effectively if the fire moves perpendicular to the stripes [23]. Although these approaches are very promising, relatively fewer studies have faced the problem at the real-world landscape [2, 24–27]. Both operational and fuel reduction management tactics are optimized at the landscape level, where spatial patterns of fuel management activities were scheduled using a heuristic optimization algorithm that was implemented in FARSITE [2]. FARSITE is a complex wildfire growth and behaviour modelling system that can simulate fire spread on real heterogeneous landscapes using spatial information on topography and fuels along with weather and wind streams [28]. Several management activities, namely prescribed burning, thinning and timber harvesting have been evaluated and compared across four landscapes with increasing fuel content [24]. The distribution of activities was achieved through optimization using SIMMPPLE (http://www.fs.fed.us/rm/missoula/4151/SIMPPLLE/index.html), a platform including fire behaviour, insect disease and climate change models designed as a management tool in landscape ecology [29]. Fire mitigation through the implementation of fuel breaks was simulated on in the Mara˜o mountain range, NW Portugal [25]. Several intensities of fuel treating stands considering also spatial vegetation density and residential priorities on a 16,000 ha study area in Oregon, US were evaluated with respect to forest health and ecological restoration [26]. The so-called minimum travel time (MTT) fire spread algorithm has also been used to simulate wildland fire spread [30]. In another study, the authors compared the relative effectiveness of thinning, a combination of thinning and burning, and solely burning on simulated stand-scale fire behavior as well as the effectiveness of three different arrangement of treatments, namely random, defensible fuel profile zones (DFPZ) [31], and strategically placed area treatments (SPLATs) [23] on a simulated area of Southern Cascade range, California [27].

Here, we focus on the problem of the systematic- in terms of mathematical modelling and analysis-placement of fire breaks as realized by fuel reduction (e.g. selective harvesting or prescribed burning) for the management of wildland fires. Besides limiting fire spread, fuel reduction is also used to restore longleaf pine forests, enhance the growth rate of young trees reducing midstorey and shrub layers, while at the same time can be used for commercial thinning of woodlands. We propose a computational two-level framework, where fire is being modeled to spread through a weighted directed network whose edge weights are the state transition probabilities of a spatio-temporal Markov Cellular Automata (CA) process [32, 33]. The particular CA model incorporates detailed GIS, landscape and meteorological data and has been proved to be robust and efficient in predicting the fire spreading behaviour in several real-world cases [32–35]. Thus, the problem of the spatial distribution of fire breaks is reduced to the problem of finding the group of nodes through which the fire spreads most rapidly. This problem is closely and straightforwardly related to the analysis of information flow on networks. For the detection of fire spread hubs, we make use of the network centrality measures such as the Betweenness Centrality and Bonacich information flow criterion [36, 37]. Our approach is illustrated through two examples: (a) an artificial forest of randomly distributed density of vegetation, and (b) a real-world case concerning the island of Rhodes in Greece whose major part of its forest was burned in 2008. Simulations, over an ensemble of lattice realizations and multiple ignition points, show the approach to be very promising as it produces statistically significantly better outcomes than the benchmark/conventional policy of fuel reduction based on the density and flammability of the vegetation.

## Materials and Methods

**Fig 1:**
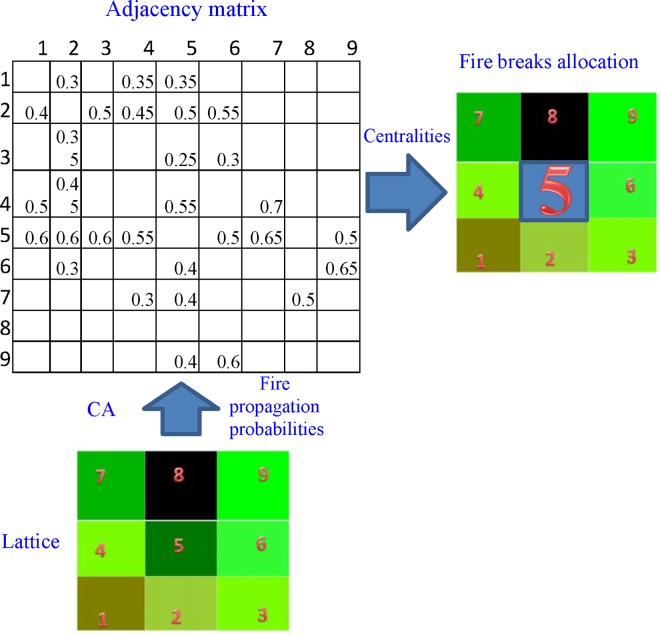
Schematic of the approach. The fire propagation probabilities are computed using a Cellular Automata model; these constitute the elements of the adjacency matrix of the lattice. Then centrality measures such as the Betweenness Centrality, Closeness and Bonacich centrality can be implemented to find the nodes (patches of land) which contribute more to the spread of the fire. (With black color is depicted an empty cell (containing no fuel). For the particular adjacency matrix shown, the most “infuental” node for the spread of the fire is the cell (node) 5.)

The proposed methodology aims at the systematic design of the spatial distribution of fire breaks for the inhibition of the propagation of wildland fires in heterogeneous forest landscapes. Fire propagation is being modeled as a random walk on a network (lattice graph of dimension NxN) with directed weighted edges; a CA model defines the transition matrix of the random walk processes [38]. Then, the spatial distribution of fire breaks is reduced to a flow information problem: that of finding a partition of network nodes that if removed from the network the propagation of fire through the network slows down the most (for a schematic see Fig 1). The terrain is tessellated into a number of small patches whose shape and size depends on the level of precision with respect to the spatial directionality (squares, hexagons etc.). Hence, the terrain is transformed into a lattice network, *G*(*V*,*E*), where *V* = {*v*_*k*_}, *k* = 1, 2, …, *N* is the set of nodes (the terrain patches), and *E* is the set of edges (links) between neighbor nodes. Each edge $e_{v_{k}\to v_{l}}$ is directed and its weight is determined by the states of the nodes *v*_*k*_,*v*_*l*_ ∈ *V* associated with it as it will be explained below. The system state over the set of the nodes (edges) is denoted by *S*(*V*) (*S*(*E*)), where *S*(*v*_*k*_) ≡ {*s*_*i*_(*v*_*k*_)} = {*s*_*ik*_}, *i* = 1, 2, …, *M* is the set of *M* state variables of the node *v*_*k*_. The state variables can be continuous (*s*_*ik*_ (⋅) ∈ *R*) or discrete (*s*_*ik*_ (⋅) ∈ *Z*) and represent both terrain and vegetation characteristics such as elevation, type and density of vegetation, moisture content, vegetation height, etc. In general, the values of state variables can change over time. In addition to the landscape characteristics, they also include a component associated to the susceptible-burning-burned states: *s*_1*k*_ = 0 if *v*_*k*_ is still susceptible to burning, *s*_1*k*_ = 1 if *v*_*k*_ is burning, and, *s*_1*k*_ = −1, if *v*_*k*_ has already been burned. Let us also denote the neighborhood of *v*_*k*_ as ${ℜ}_{v_{k}}$. The neighbourhood of each node *v*_*k*_ is defined by a set of the surrounding eight nodes (Moore-neighborhood). Thus, the system’s dynamics can be represented as a spatio-temporal Markov process of order two in space and order one in time with transition probabilities:

$$p\left(s_{1k}\left(t+1\right)=-1|s_{1k}\left(t\right)=1\right)=1$$ (1)

(the above relation implies that a node *v*_*k*_ that is burning at time *t* will be burned down at the next time step),

and,

$$p\left(s_{1k}\left(t+1\right)=1|s_{1l}\left(t\right)=1\right)=p_{v_{l}\to v_{k}}$$ (2)

where, $v_{l}\in {ℜ}_{v_{k}}$ and $p_{v_{l}\to v_{k}}=f\left(s_{ik},\;s_{il}\right)$ is the probability that the fire spreads from $v_{l}\in {ℜ}_{v_{k}}$ to *v*_*k*_ and depends only upon all the other states of *v*_*k*_, *s*_*ik*, *i* = 2, 3, …,M_, and of all the other states of its neighbors, $s_{i{ℜ}_{v_{k}},\;i=2,\;3,\;\ldots ,\text{M}}$. The transition probability $p_{v_{l}\to v_{k}}$ defines the directed weight of the spatial connection $e_{v_{l}\to v_{k}}$. In the general case, we have $p_{v_{l}\to v_{k}}\neq p_{v_{k}\to v_{l}}$, so thus the network is directed and self-contacts are not allowed, i.e. $s_{e_{v_{k}\to v_{k}}}=0$. Hence, the elements *a*_*lk*_ of the corresponding (weighted) adjacency matrix, *A*, of size *N* × *N* represent the weights of the links $e_{v_{l}\to v_{k}}$. Under the above formalism the system dynamics can be presented as a second order directed Markov field.

Having computed the adjacency matrix, centrality statistics such as the Betweenness Centrality (BC) [37] and the Bonacich information criterion [36] (see Appendix) can be used for the identification of nodes that contribute more to the fire spread through the network. The BC centrality of a node is defined as the fraction of geodesic distances (shortest paths) from all other nodes. Bonacich centrality, which is closely related to Eigencentrality (see Appendix) reflects the influence of a node on the spread of information through the network [39–41]. Here the weights of the graph, i.e. the probabilities of fire propagation $p_{v_{l}\to v_{k}}$ are inversely proportional to the distances between a given node *v*_*l*_ and its neighbors *v*_*k*_. Thus nodes with a high value of centrality are connected with other nodes by relatively short paths (involving high values of probabilities of fire propagation) and so they are central to the information flow (and therefore to the fire spread) through the network.

Under this view, we rank cells according to their centrality values from higher to lower values and we remove (i.e. cut/burn the vegetation in the corresponding land patches) the first, *N*_*e*_ ⊂ *N*, of them.

At this point, we note that for large and very large scale problems the computation of the Betweenness centralities for all nodes is computationally expensive as it requires the derivation of all shortest paths in the graph. On the other hand, the Bonacich centrality can be computed efficiently using Arnoldi’s iterative method (see Appendix).

### The artificial forest model

To illustrate the efficiency of the above measures, we first consider a simplistic case of a square lattice with Moore Neighborhood and periodic boundary conditions, where the landscape is flat, the type of vegetation is homogenous, i.e. there is just one type of vegetation (e.g. pine trees) and the density of vegetation varies continuously from 1 (corresponding to empty/burned cells) to 0 (corresponding to very dense vegetation). The state (density of vegetation) of a node *v*_*k*_ at time *t* is represented by *s*_2*k*_(*t*) ∈ [0 1]. Dynamics advance from time *t* to time *t+1* for all nodes simultaneously according to the following rules:

$$Rule\;1:\;IF\;s_{1k}\left(t\right)=1\;THEN\;s_{1k}\left(t+1\right)=-1.$$ (3)

This rule implies that a burning node at the current time step will be burned down at the next time step.

$$Rule\;2:\;IF\;s_{2k}\left(t\right)\in \left[0\;1\right)\;THEN\;IF\;s_{1{ℜ}_{v_{k}\left(t\right)}}=1\;THEN\;s_{1k}\left(t+1\right)=1\;with\;probability\;p_{b}=1-s_{2k}\left(t\right)$$ (4)

This rule implies that if a node contains fuel then if there is also a burning node at its neighborhood, it will catch fire at the next time step with probability that is proportional to the density of the vegetation. Hence, according to the above definition, if *s*_2*k*_(*t*) = 1 (meaning that the cell is empty of vegetation or has been already burned) then *p*_*b*_ = 0, while if *s*_2*k*_(*t*) = 0 (meaning that the cell contains a very dense vegetation) then *p*_*b*_ = 1.

In the random field Markov process framework, the above CA rules can be written compactly as:

$$p\left(s_{1k}\left(t+1\right)=-1|s_{2k}\left(t\right)=1\right)=1,$$ (5)

and

$$p\left(s_{1k}\left(t+1\right)=1|s_{1{ℜ}_{v_{k}\left(t\right)}}=1\right)=1-s_{2k}\left(t\right).$$ (6)

For illustrating our approach, we first considered a lattice of 50x50 cells with periodic boundary conditions. Random numbers were created by a uniform distribution in (0 1) using Matlab’s function rand. We used 100 realizations (ensembles) of randomly generated artificial forests and for each one of the realizations we created a corresponding distribution of fire breaks (randomly placed or network-based placed) and we run the simplistic CA model, until there were no burning cells. All simulations started by setting a fire at the center of the lattice.

### The case of Rhodes island, Greece

**Fig 2:**
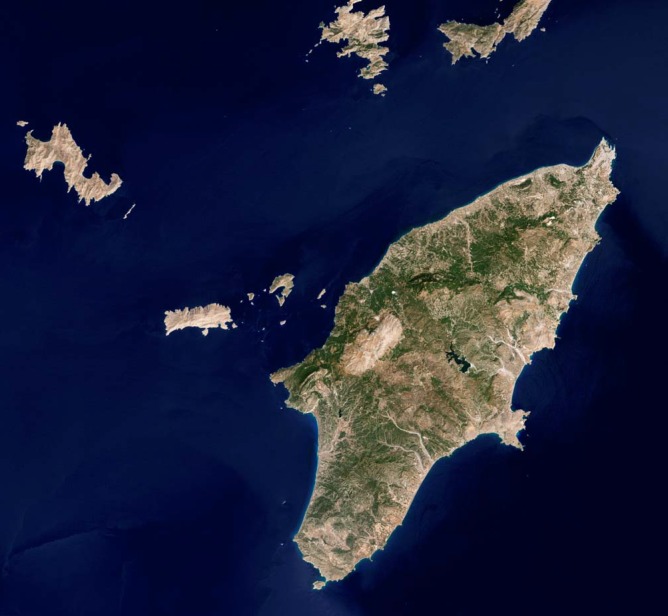
A stereoscopic image of Rhodes island, Greece (acquired from the NASA Earth Observatory (public domain): http://earthobservatory.nasa.gov/).

**Fig 3:**
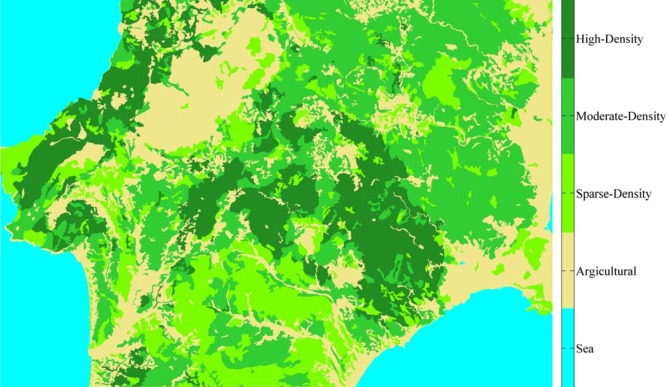
Map of the vegetation density of the area under study in Rhodes island, Greece as was before the wildfire incident of July 2008.

We applied the proposed methodology to simulate the effect of the distribution of fire breaks in the south part of Rhodes island, Greece located in the southeastern Aegean Sea. Fig 2 shows a stereoscopic image of Rhodes as acquired from the NASA Earth Observatory (http://earthobservatory.nasa.gov/IOTD/view.php?id=77079). In July 2008, a wildland fire swept through Rhodes causing significant damages. The fire occurred on 22.07.2008 and broke out at 11.40 am at the Ag. Isidoros point (Latitude: 36.15, Longitude: 27.85). Thousands of Pinus brutia trees were burned. Fig 3 depicts a map of the vegetation density of the area under study. The wind was NW 4–5 bf during the period of the incident. The consequences of the fire were 13,240 ha of burnt area and an inestimable environmental disaster.

The key and crucial element for the extension of the above approach to real-world cases is the computation of transition probabilities that produce good approximations of real fire behaviour. Here, we employed a state-of-the-art CA model that has been developed over the past few years and has proven robust and efficient in predicting the fire spreading behaviour in several real-world cases [32–35].

The CA model takes into account major macroscopic factors that determine the spread of the fire such as the vegetation density and type, wind field and detailed terrain data. For each node *v*_*k*_ we consider the following states:

$$\left\{s_{2k},\;s_{3k},\;s_{4k}\right\}=\left\{\text{impact of the type of vegetation, impact of the density of vegetation, node's elevation}\right\}$$

In a nutshell, fire dynamics (considering no wind conditions) are propagated from a node *v*_*l*_ (assuming that is burning) to its neighbors as:

$$p_{v_{l}\to v_{k}}=p_{0}\left(1+s_{2k}\right)\left(1+s_{3k}\right)f\left(s_{4k},\;s_{4l}\right).$$ (7)

Here, *p*_0_ is a nominal probability of fire spread under no wind, flat terrain, certain density and type of vegetation, and it is calculated from experimental data. The type and the density of vegetation in the area are split into a number of discrete categories. For the case of Rhodes island, the type of vegetation has been clustered into three categories (agricultural areas, pine trees, and other types of trees including shrubs), while the density of vegetation has been scaled into three categories (low, medium, high). The gain factor *f*(*s*_4*k*_, *s*_4*l*_) denotes the effect of the slope between nodes *v*_*l*_ and *v*_*k*_ and is calculated via:

$$f\left(s_{4k},\;s_{4l}\right)=\exp\left(a\theta_{s}\right)$$ (8)

where *θ*_*s*_ is the slope angle between *v*_*l*_ and *v*_*k*_ and *a* > 0 is a constant that can be adjusted from experimental data. For a square grid, the slope angle is calculated in a different way depending on whether the two neighboring nodes are adjacent or diagonal to the burning node. More specifically for an adjacent node, the slope angle reads:

$$\theta_{s}=\tan^{-1}\left(\frac{s_{4l}-s_{4k}}{l}\right),$$ (9)

where *l* is the length of the square side. For diagonal nodes the formula becomes:

$$\theta_{s}=\tan^{-1}\left(\frac{s_{4l}-s_{4k}}{l\sqrt{2}}\right)$$ (10)

Note that the gaining factors to in Eq 7 can exceed 1. For example the factor related to the slope by Eq 8 can exceed 1 for positive slopes *θ*_*s*_. However, the final probability is constrained between 0 and 1.The effect of wind can be also considered by multiplying Eq 7 appropriately (see [32]).

The parameter values of the above model are given in Table 1. Keeping the same values of the model parameters as the ones found through optimization for the wildland fire that occurred in Spetses island in Greece in 1990 [32], the model predicted quite well the dynamics of the large scale wildfire that occurred in the mountain of Parnitha, Greece in 2007, one of the most catastrophic fire incidents in Greece over the last 50 years [33], and that of wildland fire which devastated Rhodes in 2008 [34]. More specifically, for the case of Spetses island the wildfire burned an area of about 590 ha in 11 hours; the CA simulator over 100 runs resulted to an average of 540 ha (std: 100 ha) burned area (almost equivalent to the actual burned area) in 11.3 (std: 2.5) hours [32]. For the case of the wildfire in Parnitha, Athens, the CA simulator resulted to an almost equivalent spatio-temporal pattern to the actual evolution of the wildfire that burned 5,600 ha in 3 days [33]. We note here that these areas belong to the same bio-geo-climatic domain. Furthermore both islands (Rhodes and Spetses) contain the same type of vegetation.

**Table 1:** Parameter values for the CA model.

Parameter	Value	Effect of Density (*s*_3*k*_)		Effect of Type (*s*_2*k*_)	
*p*_*o*_	0.58	Sparse	-0.4	Agricultural	-0.3
*a*	0.078	Moderate	0	Other types of trees including shrubs	0
		Dense	0.3	Pines	0.4

**Fig 4:**
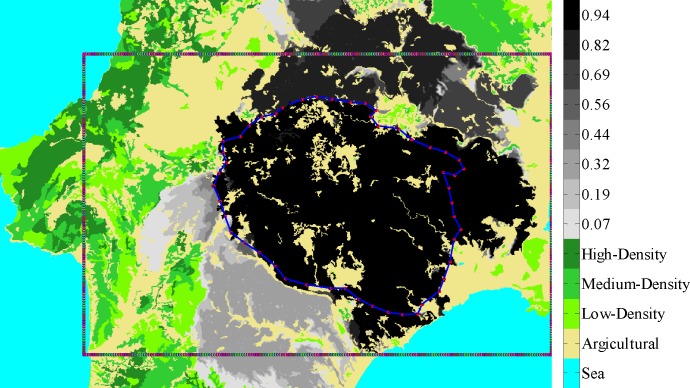
The “expected” map of relative burning frequencies for the case of Rhodes island, Greece. This was constructed using the CA model over 100 simulations all starting from the ignition point of the real incident of 22.07.2008 (Ag. Isidoros: Latitude: 36.15, Longitude: 27.85)**;** the outline of the burned area of the real incident is also shown.

The area was tessellated using a two dimensional grid. Each cell of the grid defines a node representing a small square patch of land, thus offering eight possible directions of fire spread. The side of the square was equal to ten meters. Thus, the size of the adjacency matrix was 1260000 x 1260000 reflecting the area of interest defined by the rectangular shown in Fig 4.

### Performance evaluation

In order to access the effectiveness of the proposed approach, we measured the hazard intensity *R*, with respect to the density of fire breaks *d*_*f*_, defined as:

$$R\left(d_{f}\right)=\frac{1}{n_{r}}\sum_{i=1}^{N_{r}}\frac{n_{b}\left(i\right)}{n_{v}}$$ (11)

where $d_{f}=\frac{n_{fb}}{n_{v}}$ is defined as the ratio of the number of fire breaks *n*_*fb*_, and the total number of nodes that contain flammable vegetation *n*_*v*_. The parameter *n*_*r*_ denotes the number of simulations for a given initial condition, while *n*_*b*_ (*i*) denotes the total number of burned nodes of the i-th simulation. For the artificial forest model, which is characterized by random distribution of densities in a flat terrain, the proposed approach is tested against the benchmark of random distribution of fire breaks, while for the real case of Rhodes island, the effectiveness of the approach was tested against the conventional fuel reduction treatment based on the density and flammability of the vegetation. For this case, the conventional tactic was implemented as follows: within the area of interest, nodes were sorted from high vegetation densities of pine trees to high densities of other types of trees including shrubs, to moderate vegetation densities of pine trees followed by other types of trees including shrubs, to sparse vegetation densities of pine trees followed by other types of trees including shrubs; then the first *d*_*f*_ percent of the sorted nodes was selected to be removed. In both approaches (proposed and benchmark/conventional), the removal of two neighboring (adjacent) nodes was not allowed even if were highly ranked to avoid the formation of clusters of empty zones.

We simulated *n*_*r*_ = 3,200 randomly placed ignitions (as e.g. in [42]) letting the simulator to run until the fire stops. Statistically significant differences for a given *d*_*f*_ (discretized at levels of 0.1) between the proposed and conventional tactic were computed by implementing the t-test on the outcomes (*R*-statistic defined in Eq 11) of the simulations with a threshold set at *a* = 0.05.

It should be noted that as the model is stochastic, simulations will generally result in different *n*_*b*_s (number of burned cells). For this reason, we have chosen to run the model *n*_*r*_ = 100 times with the same ignition point (the point where the fire actually started) and depict all simulations by mapping the burning frequency of each cell in a gray-scale mode. Hence, for each cell we calculated the frequency of burnings out of the 100 simulation runs. Then, we created the corresponding histogram of average number of burnings for the area under study using 2^4^ bins. Finally, we assigned—in descending order—2^4^ levels of gray (from 2^8^ to 2^4^ with a discretization step of 2^4^) to the bins. We applied the proposed approach for the risk management of a particular area (defined by the rectangular shown in Fig 4) using the Bonacich measure with a *β* = 0.5 (choosing different values of *β* between 0 and 1 resulted to equivalent results). The performance of the methodology was compared to the benchmark approach of conventional fuel reduction.

## Simulation Results

### The artificial forest problem

**Fig 5:**
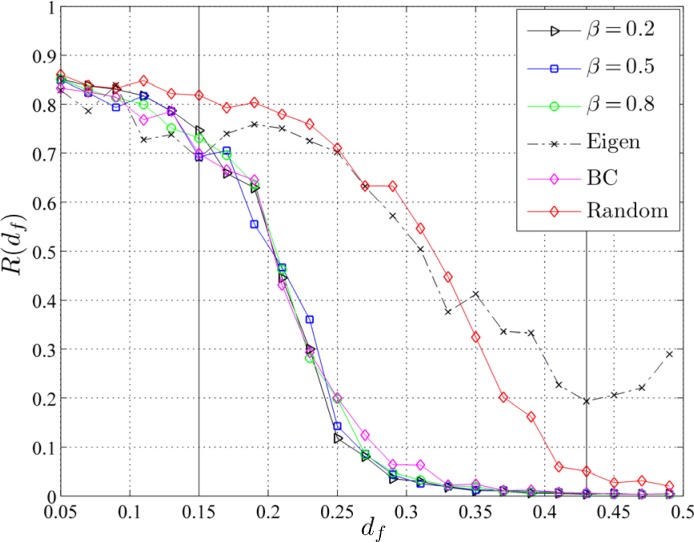
The simple case of an ensemble of artificial forests. Hazard intensity *R*(*d*_*f*_) as obtained using the Bonacich (with *β* = 0.2, 0.5, 0.8), BC, Eigencentrality and the random distribution of fire breaks. The shaded area marks statistically significant differences between Bonacich and BC vs. random distributions as computed by the t-test (at *a* = 0.05).

Fig 5 depicts the diagram of the hazard intensity, *R*(*d*_*f*_) for the centrality measures described at the Methods and Material section. The results obtained with the random distribution tactic are also shown for comparison purposes. The shaded area shows the range of fire breaks densities where differences between the Bonacich and the BC-based are statistically significant from the random-based distribution. As it is clearly shown, the proposed approach based on the Bonacich criterion for various values of *β*s (0.2, 0.5, 0.8) and the BC outperforms significantly the random-based distribution of fire breaks. The Bonacich criterion for the above mentioned range of *β* values gives equivalent results with the BC measure.

For the Bonacich and the BC-based approach there is a relatively sharp phase transition from high to low hazard intensities (*R*s) around *d*_*f*_ = 0.22 while for the random-based distribution the phase transition occurs around *d*_*f*_ = 0.32. On the other hand, Eigencentrality [36, 37] performs poorer even when compared to the random-based distribution, thus resulting to higher fire hazard for bigger densities of fire breaks (see Fig 5). This is in line with other studies reporting that the Eigencentrality criterion may not produce meaningful results [36] (see Appendix).

### The real case of Rhodes island, Greece

The resulting “expected” map of relative burning frequencies as derived using the CA simulator is shown in Fig 4. For comparison purposes we have overlaid the outline of the actual burned area. As it is shown, the “expected” burned area predicted by simulations is close enough to the real one: if one takes the upper 85% then the actual and simulated burned areas almost coincide.

**Fig 6:**
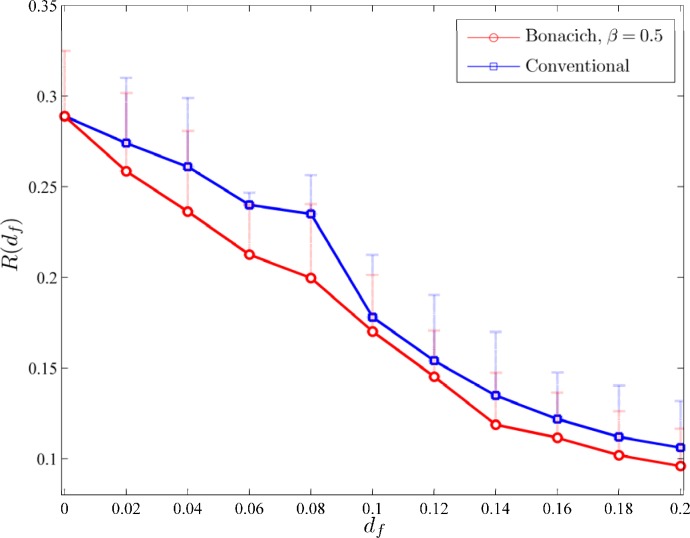
The case of Rhodes island, Greece. Hazard intensity *R*(*d*_*f*_) using the proposed (circles) and conventional (squares) distribution of fire breaks, as computed over *n*_*r*_ = 3,200 simulations starting from equally-numbered ignition points. 90% percentiles are also illustrated with dotted bars. Statistically significant differences between distributions as computed by the t-test (at *a* = 0.05) arose for all values of *d*_*f*_ > 0.

Fig 6 depicts the diagram of average *R*(*d*_*f*_) over *n*_*r*_ = 3,200 runs (multiple ignition points). The results obtained with the conventional treatment tactic are also shown for comparison purposes; 90% percentiles of the burned area are also illustrated with bars. The t-test (with a threshold set at *a* = 0.05) showed that the mean *R*(*d*_*f*_)s of the two approaches differ significantly for all the values of *d*_*f*_ > 0: the proposed approach results to lower wildland hazards. In particular, for *d*_*f*_ ≥ 0.14 the proposed approach results to a fire hazard at least 10% lower than the conventional approach. Furthermore, for all the range of *d*_*f*_s the proposed approach results to significantly lower maximum values of burned areas. For example for *d*_*f*_ = 0.14 the proposed approach results to ~15% lower maximum (at the level of 90% percentile) value of burned area.

At this point it is interesting to pinpoint the differences in the distributions of fire breaks with respect to the type and density of vegetation between the proposed approach and the conventional strategy. For example for *d*_*f*_ = 0.14, the proposed (vs. conventional) approach allocates (a) ~15% (~7.5%) of the fire breaks in nodes with sparse, ~15% (~18%) in nodes with moderate, ~14.5% (~19%) in nodes with dense density of *pine trees*, and, (b) ~12% (~0%) of the fire breaks in nodes with sparse, ~12% (~14%) in nodes with moderate, and ~21% (~26%) in nodes with dense density of *other types of trees including shrubs*. Similar outcomes hold for all values of *d*_*f*_s. The above results reveal that targeting management activities mainly on the dense vegetation is not always so effective. It may be needed to consider managing patches with sparse density of vegetation which however may act as hubs for the spread of the fire.

## Discussion

It has been recognized that fire is an essential element of forest renewal, enhancing the growth of young and old trees and improving plant resources [43]. On the other hand, uncontrolled wildfires have been the cause of numerous irreversible environmental damages with serious negative ecological and socio-economic consequences including loss of human lives, flora and fauna bio-diversity and rare-species destruction, habitant fragmentation, floods, loss of timber harvest capability, economic loses in the tourism sector, air pollution and climate change. Hence, one of the most challenging land management revolves around the design and implementation of efficient wildland fire-prevention as it has proven to be much safer and cost-effective than reactive responses to unplanned emergency fires [44, 45].

The systematic-in terms of mathematical modelling and analysis–quantification of the fire spread dynamics is of outmost importance towards the risk assessment of a potential outbreak. However, due to the inherent complexity of such a phenomenon deploying at different time and space scales, risk assessment is far from simple.

The computational approach proposed here is devoted to the design of the distribution of fire breaks with the aid of complex network theory and detailed CA modeling. The proposed approach is based on the concept of centrality, a key statistical measure for the evaluation of information flow through complex networks. The approach involves the construction of the adjacency matrix whose elements are the strengths (weights) of the fire propagation. The CA-based model used to calculate the transition probabilities of fire spread among the land patches can deal with spatial heterogeneity in both the fuel and landscape characteristics and can take as input local meteorological data (even in real time). It has been proven to be robust and efficient in predicting the fire spreading dynamics both in space and time in several real-world large-scale wildland fires [32–35].

The proposed approach is here illustrated through: (a) a simplistic lattice configuration which encompasses a single type of vegetation with randomly varying density and (b) a wildfire occurred in the island of Rhodes Greece in 2008. Our approach is compared with the conventional tactic of fuel reduction (e.g. selective harvesting, prescribed burning) based on density and flammability of the vegetation. It is shown, through simulations, that the proposed approach outperforms the conventional forest management practice.

The methodology can be combined with contemporary forestry management that makes use of either properly designed surface fires to restore longleaf pine ecosystems [17, 46] or tree cutting/harvesting that can under certain restrictions enhance forest sustainability. For our illustrations, the comparison was made in the absence of wind. However, the effect of the direction and the speed of wind can be accommodated in a straightforward way [32]. It would be also interesting as a future work to compare the efficiency of the proposed approach with other proposed strategies [2, 24–27]. For example compartmentation of the landscape, i.e. the creation of bulkheads patterns enclosing forest patches has been proposed [22]. However this approach, while effective may lead to significant distortion of the underlying landscape. To address this issue a creation of random distribution of firebreaks in landscape patterns that appear somewhat natural has been suggested [47]. In recent years, various studies have turned their attention towards the optimal distribution of fire breaks within a multi-objective decision-making framework [2, 48– 50]. The effect of four basic patterns of fuel management has been studied including regularly spaced, dispersed, clustered and random that were scheduled using heuristic optimization techniques with stand density and harvest volume goals [2]. The study site was the Grand River Basin in northeastern Oregon. The performance of the four resulting patterns was examined using FARSITE. The number of simulations was limited due to the computational cost. Simulations showed only minor differences between patterns with marginal reduction in the level of wildfire hazard [2]. The problem has been presented as a combinatorial simulation-optimization problem for minimizing the fire-risk subject to a limit on the total area of fuel breaks [48]. The model was tested on a forest of 220,000 ha in north-western Ontario. Optimized results were compared with the random as well as the conventional approach that selects the highest ranking cells for fuel-treatment until the limit of the total area of fire breaks is reached. It has been shown that while the method outperforms significantly the random approach, it performs comparably with respect to the conventional approach having a fire-risk approximately 5% lower than the latter. Simulated annealing has also been implemented to schedule fuel treatment across time and space [49]. This approach was applied to a 14,000-ha study landscape located on the west side of the Bitterroot Valley in Montana. The authors showed that optimized solutions outperform the randomly placed ones. An important obstacle that all the above studies indicate is the computational intensity of the stochastic optimization approach requiring a large numbers of iterations, while the “best” global optimal solution is not guaranteed.

Compared to the above approaches our methodology addresses a systematic, straightforward way for the spatial allocation of fire breaks and importantly for the identification of nodes that may act as hubs of the fire spread. The importance of the management of such sparse-density patches has not been highlighted yet in the literature.

## Conclusions

Based on complex network theory, we proposed a systematic methodology for assigning the spatial locations of fire breaks to inhibit wildfire hazard in heterogeneous forest landscapes. The partition of the barriers is achieved using a two-level algorithm which involves: (a) the construction of a weighted lattice graph, where the weights correspond to transmission probabilities of fire propagation, (b) the identification of cells (network nodes) that contribute more to the fire spread through the network; these correspond to nodes with high centralities. The edge weights are computed using the relations of fire propagation probabilities through a detailed Cellular Automata fire propagation model which takes into account detailed terrain, meteorological data as well as vegetation characteristics such as type, density, flammability and moisture content. The efficiency of the approach was compared against the typical practice of fuel reduction (selection harvesting) based on density and vegetation flammability. The site of study was Rhodes island, Greece which suffered from a large wildland fire back in 2008; 3,200 simulations starting from equally numbered randomly placed ignitions were considered. Simulations results showed that the proposed approach results in statistically significant lower hazards. An important outcome of the analysis is that the effective management of patches of sparse density of vegetation that act as hubs of fire spread is the key in reducing the hazard. To this end, we should point out that such an approach (as other similar approaches) should not be considered as a “one-size-fits-all” one and/or as a stand-alone strategy [4]. The appropriateness and the specifics of fuel treatment for wildland prevention remains a subject of debate among scientific community [51–53]. Questions related with the way treatments (such as reduction of tree density, thinning and prescribed burning) should be carried out need to be answered. Furthermore, important issues and restrictions related to the effects of fuel reduction management on the forestry ecosystem (e.g. such as its influence to biodiversity) have to be taken into account [26, 54–58].

## Appendix

### Centrality measures

Several measures have been proposed for the assessment of the information centrality in networks such the Betweenness centrality (BC), the closeness centrality (CC), the Bonacich centrality (BC) and the Eigen-centrality (EC) [36, 38].

The BC of node *v*_*k*_ is defined as:

$$BC_{v_{k}}=\sum_{l\neq k\neq m}\frac{n_{v_{l}v_{m}}^{v_{k}}}{n_{v_{l}v_{m}}^{}}$$ (12)

$n_{v_{l}v_{m}}^{v_{k}}$ is the number of shortest paths between nodes *v*_*l*_ and *v*_*m*_ passing from *v*_*k*_, and $n_{v_{l}v_{m}}^{}$ is the number of the shortest paths (as derived by the sum of the edge weights) between *v*_*l*_ and *v*_*m*_. Here, the weights of the network links should be inversed so that long walks count to slow spread directions, while, short walks count for fast spread directions.

The CC of node *v*_*k*_ is defined as the inverse of the sum of geodesic distances (i.e. the shortest paths) from node *v*_*k*_ to all other nodes in the network:

$$CC_{v_{k}}={\left(\sum_{m=1,k\neq m}^{N}d_{v_{k}v_{m}}^{}\right)}^{-1}$$ (13)

$d_{v_{k}v_{m}}^{}$ is the geodesic distance from *v*_*l*_ to *v*_*m*_.

The Eigencentrality of node *v*_*k*_ corresponds to the *k-th* component of the eigenvector related to the largest eigenvalue of the adjacency matrix *A*. A node with a high Eigencentrality is adjacent to nodes possessing also high scores of eigencentrality [40]. For very large scale directed weighted networks where *A* is asymmetric one can employ Arnoldi’s iterative method for extracting a low dimensional upper Hessenberg matrix whose eigenvalues of provide approximations of the outermost spectrum of the full matrix [59]. However for directed graphs, Eigencentrality may not produce meaningful results [36]. An extension of Eigencentrality to directed weighted graphs comes from the Bonacich measure defined as the *k-th* component of [36]:

$$x={\left(I-\frac{\beta }{\lambda_{\max}}{Α}^{T}\right)}^{-1}e,$$ (14)

where ***e*** is a vector of ones and *λ*_***max***_ is the largest eigenvalue of *A* (that can be computed through the Arnoldi eigensolver [59]). The notion of Bonacich centrality is that the power of a node depends on the power of the adjacent nodes. The parameter *β* can be regarded as an attenuation factor. Positive values of *β* give more weight to powerful connections while negative values to less powerful connections. Small values of *β* give more weight to the local network structure while larger values give more weight to the wider network structure [41]. For *β* = 0, the above expression reduces to the degree centrality, while for *β* = 1 it reduces to the standard Eigencentrality.
